# Alginate Hydrogel Microtubes for Salivary Gland Cell Organization and Cavitation

**DOI:** 10.3390/bioengineering9010038

**Published:** 2022-01-15

**Authors:** Matthew Jorgensen, Pujhitha Ramesh, Miriam Toro, Emily Evans, Nicholas Moskwa, Xulang Zhang, Susan T. Sharfstein, Melinda Larsen, Yubing Xie

**Affiliations:** 1 College of Nanoscale Science and Engineering, SUNY Polytechnic Institute, 257 Fuller Road, Albany, NY 12203, USA; mattjorgensen@gmail.com (M.J.); rameshp@sunypoly.edu (P.R.); torom@sunypoly.edu (M.T.); EEvans@sunypoly.edu (E.E.); zhangx3@sunypoly.edu (X.Z.); ssharfstein@sunypoly.edu (S.T.S.); 2 Department of Biological Sciences, State University of New York, University at Albany, 1400 Washington Ave., Albany, NY 12222, USA; nmoskwa@albany.edu (N.M.); mlarsen@albany.edu (M.L.); 3 RNA Institute, State University of New York, University at Albany, 1400 Washington Ave., Albany, NY 12222, USA

**Keywords:** alginate, hydrogel, microtube, 3D culture, salivary gland, organoids, cell–cell interaction, tissue engineering

## Abstract

Understanding the different regulatory functions of epithelial and mesenchymal cell types in salivary gland development and cellular organization is essential for proper organoid formation and salivary gland tissue regeneration. Here, we demonstrate a biocompatible platform using pre-formed alginate hydrogel microtubes to facilitate direct epithelial–mesenchymal cell interaction for 3D salivary gland cell organization, which allows for monitoring cellular organization while providing a protective barrier from cell-cluster loss during medium changes. Using mouse salivary gland ductal epithelial SIMS cells as the epithelial model cell type and NIH 3T3 fibroblasts or primary E16 salivary mesenchyme cells as the stromal model cell types, self-organization from epithelial–mesenchymal interaction was examined. We observed that epithelial and mesenchymal cells undergo aggregation on day 1, cavitation by day 4, and generation of an EpCAM-expressing epithelial cell layer as early as day 7 of the co-culture in hydrogel microtubes, demonstrating the utility of hydrogel microtubes to facilitate heterotypic cell–cell interactions to form cavitated organoids. Thus, pre-formed alginate microtubes are a promising co-culture method for further understanding epithelial and mesenchymal interaction during tissue morphogenesis and for future practical applications in regenerative medicine.

## 1. Introduction

Research in therapeutic tissue engineering for salivary gland regeneration has demonstrated that a tremendous amount of discovery is still needed before we can successfully replicate the in vivo environment within tissue constructs [1,2]. A sustainable and reproducible method for developing functional salivary gland tissue is needed to address both xerostomia, or the feeling of dry mouth, which is a common clinical symptom arising from low saliva output, and hyposalivation [3]. The most common conditions in which hyposalivation and xerostomia occur include: Sjögren’s syndrome, diabetes, radiotherapy for head and neck cancers, salivary gland cancers, side effects due to medication, and aging [3,4,5]. Since Sjögren’s syndrome is an autoimmune disorder, a therapeutic method involving cells that will not be recognized by an overactive immune system will be necessary. Radiotherapy, which causes irreversible damage to the acini found in the salivary gland, will require some form of tissue transplantation after the fractionated radiation therapy (i.e., multiple treatments over time) has been completed and the body has healed [5]. With salivary gland cancers of the parotid, there is often a partial or total parotidectomy, thus making desirable the transplantation of tissues or cells to replace what was removed. Possible therapeutic approaches include the transplantation of stem cells either with or without scaffolds or the promotion of differentiation of endogenous progenitor cells [5]. In the general population, simple, age-related factors account for many of the salivary gland changes that occur during a person’s lifetime [6]. Additionally, as a person ages, the chances of needing some form of medication increases, and these medications often have side effects, such as anticholinergic activity, that cause hyposalivation. Each condition presents its own unique challenges for proper development of therapeutics. Salivary gland organoid models recapitulate cellular organization, in vivo-like cell–cell interactions, and specific disease state parameters enabling many assays for assessing defective cell–cell interactions or evaluating the effectiveness of specific therapeutics. Salivary gland organoids also have the potential to be transplanted into diseased glands in cell-based therapies.

Resident stem/progenitor cells maintain tissue homeostasis and can be activated to induce regenerative or repair processes [7]. When encountering extracellular signals, stem cells can undergo self-renewal or differentiation into specific cell types. There are two major cell types in the salivary gland: “parenchymal” or epithelial cells, which have the ability to self-renew and differentiate into epithelial cell lineages found within salivary gland tissue, [8] and “stromal” or mesenchymal cells, which can contact epithelial progenitor cells directly to guide acinar cell differentiation pathways [9]. Each of these cell types are dependent on cell–cell communication through junction molecules [10] that facilitate organization into functional salivary gland tissue. To facilitate engineering of organoids, it is of paramount importance to understand epithelial–mesenchymal cell interactions and to identify signaling required to induce salivary gland tissue morphogenesis.

There are multiple platforms for evaluating cell–cell interactions. Many current epithelial and mesenchymal cell co-culture models primarily use Transwell^®^ inserts. For example, primary salivary gland epithelial cells were cultured on the Transwell^®^ membrane, and mesenchymal stem cells (MSCs) were cultured in the lower chamber, or vice versa, resulting in the MSCs adopting a salivary gland epithelial phenotype [11,12,13,14]. These co-culture models provide indirect epithelial–mesenchymal cell interaction, which can only induce 2D morphogenesis. However, direct epithelial–mesenchymal cell interaction is needed to facilitate 3D branching [15]. Three-dimensional salivary gland co-cultures have been explored by co-culturing mouse salivary gland progenitor cells and MSCs in Matrigel [9,16,17,18] and co-culturing SIMS salivary gland epithelial cell lines and NIH 3T3 fibroblasts on electrospun nanofibers [19], which provide nanotopographical cues for salivary epithelial cell organization [3,20]. Mouse submandibular salivary gland (SMG) organ explants grown on top of polyacrylamide hydrogels showed that the soft substrate with physiological compliance supported normal branching morphogenesis and acinar cell differentiation, while stiff substrates perturbed tissue architecture and epithelial acinar cell differentiation [21]. The effect of substrate compliance on salivary gland cell organization was also demonstrated using blended electrospun elastin/PLGA nanofibers with increased compliance, which supported SIMS cell cluster formation [22]. Similarly, using poly (lactic co glycolic acid)/poly (glycerol sebacate) (PLGA/PGS) nanofibers with increased compliance supported better growth of SIMS salivary gland epithelial cells, where increased expression of tight junction proteins and apicobasal polarization of SIMS cells were observed [19]. In addition, curved nanofiber substrates promoted SIMS cell polarization and maximized the height of the monolayer of SIMS cells, similar to that seen in 3D Matrigel cultures [23]. All of these studies highlight the importance of co-culturing mesenchymal cells with epithelial cells and indicate that using a scaffolding material that better resembles in vivo compliance and curvature will be more desirable for self-organization of salivary gland cells. However, methods have not been developed to produce large numbers of complex stereotyped organoids that contain both mesenchymal and epithelial cell types.

In this study, we examined the feasibility of using alginate hydrogel microtubes to provide a compliant environment with curvature as a bioengineering approach to facilitate 3D salivary gland epithelial–mesenchymal cell interactions and cellular organization. Alginate is biocompatible and widely used in tissue engineering and cell therapy applications [24,25,26,27,28]. Alginate hydrogel microtubes have shown great potential for stem cell expansion and differentiation and heterotypic hepatic organoid formation [29,30,31,32,33]. These microtubes provide an easy-to-handle 3D culture system that permits control and monitoring of cell aggregate formation while preventing the loss of these cell aggregates or organoids during medium changes [34]. Using mouse salivary gland ductal epithelial SIMS cells as the epithelial model cells and NIH 3T3 fibroblasts or primary E16 salivary mesenchyme cells as the stromal model cells, we examined the self-organization and epithelial–mesenchymal cell interactions in the microenvironment of hydrogel microtubes. Our results demonstrate that alginate hydrogel microtubes can provide a platform for the reproducible production of stereotyped, cavitated salivary gland organoids for the study of epithelial and mesenchymal cell interaction and tissue regeneration.

## 2. Materials and Methods

### 2.1. Materials

Sodium alginate and calcium chloride dihydrate (CaCl_2_·2H_2_O) were purchased from Sigma-Aldrich (St. Louis, MO, USA). Three mL disposable syringes were purchased from Becton Dickinson (Franklin Lakes, NJ, USA). Some 17 gauge and 22 gauge blunt-tip needles were purchased from Hamilton Company (Reno, NV, USA). Multiple gauged premium Kanthal wires were purchased from Amazon or Walmart. 

### 2.2. Needle-in-Needle Microfluidic Device

A needle-in-needle microfluidic device (Device 17–22) was fabricated by precise placement of a needle (22 gauge) within another needle (17 gauge) through the use of properly sized Kanthal wires wrapped in a shape around the inner needle, allowing for approximate centering of the inner needle to the outer needle as described previously [34]. This coiled feature allowed for additional mixing of the sodium alginate solution, if needed, before introduction into the CaCl_2_ solution. 

### 2.3. Microfluidic Fabrication of Alginate Hydrogel Microtubes

A 100 mM CaCl_2_ solution (core fluid), 6% sodium alginate solution (sheath fluid), and a separate 100 mM CaCl_2_ solution (collecting fluid) were prepared in 0.9% NaCl solution, followed by autoclave sterilization at 121 °C for 15 min. The sheath fluid (6% sodium alginate) was introduced through tubing connected to the outer needle while the core fluid (100 mM CaCl_2_) was introduced to flow through the inner needle of the needle-in-needle microfluidic device, where each fluid was driven by a NE 1000 syringe pump (New Era Pump Systems Inc., Farmingdale, NY, USA) using predetermined flow rates for optimal microtube formation as described previously [34]. Based on our previous studies, the inner flow rate of CaCl_2_ at 2.50 mL/min was chosen for the needle-in-needle microfluidic device 17–22 [34]. Then we varied the alginate flow rate (1.75–3.00 mL/min) to determine the alginate flow rate that gives rise to alginate hydrogel microtubes with minimal variation of the hydrogel microtube inner diameter and with a wall thickness around 200 µm, which is strong enough for handling and thin enough to allow diffusion of nutrients into the microtubes. Through the extrusion process, these two fluids were directed into a collecting pool of CaCl_2_ solution contained within a 50 mL conical tube. When the sodium alginate solution converged with the CaCl_2_ solution, immediate alginate crosslinking occurred, creating an alginate hydrogel microtube. Upon introduction into the conical tube filled with the collecting fluid, the “liquid rope-coil effect” occurred [35]. This effect allowed for an organized stack of alginate hydrogel microtubes for ease of storage and handling in future experiments (Figure S1). These pre-formed alginate microtubes were then stored at 4 °C in 50 mL conical tubes filled with 100 mM CaCl_2_ until needed for cell culture experiments (Figure S1a). 

### 2.4. Cell Culture 

Mouse NIH 3T3 fibroblasts [36] and SIMS ductal salivary gland epithelial cells [37] were grown in culture media composed of high glucose Dulbecco’s Modified Eagle’s Medium (DMEM) (Sigma-Aldrich), supplemented with 10% fetal bovine serum (FBS) (Sigma-Aldrich), 1% penicillin-streptomycin (pen/strep, 10,000 units penicillin and 10 mg streptomycin per mL, Sigma-Aldrich). Cells were cultured in a 37 °C, 5% CO_2_ humidified incubator and subcultured by trypsinization and replating every 2–3 days for NIH 3T3 fibroblasts or every 4–5 days for SIMS cells. The medium was typically replaced every other day. 

### 2.5. Isolation and Culture of Mouse Primary E16 Salivary Mesenchyme Cells

Mice used to source salivary glands were embryonic day 16 (E16) timed-pregnant CD-1 female mice from Charles River Laboratories (Wilmington, MA, USA). The care and handling of mice were carried out in accordance with the National Institutes of Health Guide for the Care and Use of Laboratory Animals, and protocols were approved by the Institutional Animal Care and Use Committee (IACUC) of the University at Albany, State University of New York. Primary E16 mesenchyme cells were isolated from mouse SMGs, as previously described [9,18,38,39] and then grown in culture medium composed of Gibco DMEM/F-12 (Thermo Fisher Scientific, Grand Island, NY, USA), without HEPES or phenol red, supplemented with 10% FBS, 1% pen/strep (Sigma Aldrich). Cells were cultured in a 37 °C, 5% CO_2_ humidified incubator and subcultured every 2–3 days for no more than 2 or 3 passages. The medium was typically replaced every other day.

### 2.6. Cell Injection and Co-Culture in Pre-Formed Alginate Hydrogel Microtubes

For optimization of the epithelial to stromal ratio, SIMS (5 × 10^6^ cells/mL) and NIH 3T3 cells (5 × 10^6^ cells/mL) were mixed at a ratio of 1:5, 5:1, or 5:5 to obtain 1 mL of cell mixture with a final cell density of 3 × 10^6^ cells/mL medium. For co-culture of SIMS cells with E16 mesenchyme cells at a ratio of 1:5, 0.1 mL SIMS (5 × 10^6^ cells/mL) and 0.5 mL E16 cells (5 × 10^6^ cells/mL) were mixed with 0.4 mL culture medium. The cell mixture in each condition was then transferred to a syringe with a 22 gauge blunt needle attached to it. The syringe was positioned to allow insertion of the attached needle into the open end of the pre-formed alginate hydrogel microtubes (Figure S1b), and the cell mixture was injected into the microtube until reaching the end of the microtube. After cell injection, each hydrogel microtube was picked up with a pair of tweezers (that were sterilized through dipping in absolute ethanol followed by flaming) and placed into a well of a 6-well plate. After cells settled for 20 min, the open ends of the microtube showed empty space (i.e., no cells) for about 10 mm. Although the empty space might be caused by cells washing out with fluid motion at the end of the microtube, we did not detect many cells in the medium. Afterwards, cell clusters did not “escape” from the microtubes during medium changes. The microtubes were cultured with either DMEM (SIMS:NIH 3T3 co-culture) or DMEM/F-12 (SIMS:E16 co-culture) media, supplemented with 10% FBS, 1% pen/strep at 37 °C, and 5% CO_2_ humidified air atmosphere. Medium was changed every other day. Ibidi 8-well µ-Slide cell culture chambers (Ibidi, Fitchburg, WI, USA) were used for co-culture experiments at 1:5 epithelial to stromal ratio as the 2D control, where each µ-Slide well contained approx. 2.4 × 10^4^ total cells with 300 µL of culture medium, which was replaced every day.

### 2.7. Optical Imaging

Pre-formed alginate hydrogel microtubes were imaged using an inverted microscope (Nikon Eclipse TS100, Micro Video Instruments, Avon, MA, USA) equipped with a Retiga 2000R digital camera and QImaging software. Optical images of microtubes were analyzed using NIS-Elements D software, and inner and outer diameters of the microtubes were calculated as the mean ± standard deviation. The wall thickness was calculated by subtracting the inner diameter from outer diameter and dividing by 2. Cell growth in hydrogel microtubes was monitored daily using the Nikon TS100 inverted microscope. Optical images were captured using a 4x objective lens on days 1, 4, and 7.

### 2.8. Live and Dead Cell Assay

For cells cultured in hydrogel microtubes, a small section of alginate hydrogel microtube containing cell aggregates/clusters was removed from the 6-well plate and placed on an Ibidi 18-well µ-Slide. A total of 20 µL of culture medium was then added followed by 6 µL of Abcam Live and Dead Cell Assay stain solution. Slides were then placed in an incubator for 20 min at 37 °C. Observations and images were taken on a Leica Confocal Microscope TCS SP-5 controlled by LAS-AF software using 20x objective lens (Leica Microsystems, Mannheim, Germany), with excitation/emission of 488 nm/519 nm for live cells and 561 nm/575 nm for dead cells, respectively.

### 2.9. Immunocytochemistry Analysis

On days 1, 4, and 7 of culturing, cell clusters were released from the microtube by dissolving the alginate hydrogel using 1.6% sodium citrate solution (Sigma-Aldrich) for 1–5 min for microtubes cultured without supplemental CaCl_2_ or up to 10 min for microtubes cultured with 25 mM CaCl_2_. All cell clusters released from hydrogel microtubes that were collected in microcentrifuge tubes or cells cultured in the µ-Slide cell culture chamber were then rinsed with 1x phosphate buffer saline (PBS, Corning Inc., Corning, NY, USA) and immediately fixed with 4% paraformaldehyde (PFA, Sigma-Aldrich) in PBS for 30 min at room temperature. Samples were rinsed with 1x PBS-Tween (0.1% Tween 20 in 1x PBS, Fisher Thermo Scientific, Waltham, MA, USA) three times and permeabilized using 0.1% Triton X-100 (Sigma-Aldrich) in PBS for 15 min at room temperature. After rinsing again with PBS-Tween three times, samples were incubated in blocking solution, 5% bovine serum albumin in PBS (BSA, Sigma Aldrich), at 37 °C for 30 min. After removal of excess blocking solution, primary antibodies prepared in blocking solution containing DAPI (4′,6-diamidino-2-phenylindole, Sigma-Aldrich) were added to samples and incubated overnight at 4 °C on a rocker. Primary antibodies were as follows: Alexa Fluor^®^ 647-conjugated EpCAM (1:100, BioLegend, San Diego, CA, USA) and Alexa Fluor^®^ 488-conjugated vimentin (1:100, Cell Signaling Technology, Danvers, MA, USA). The following day, samples were rinsed using 1x PBS-Tween three times. Cell cluster samples were removed and placed on Ibidi 18-well µ-Slide cell culture chambers. Samples were observed and imaged using a Leica Confocal Microscope TCS SP-5 controlled by LAS-AF software using 10x objective and 63x oil immersion objectives (Leica Microsystems, Mannheim, Germany) at excitation/emission of 647 nm/671 nm, 488 nm/519 nm, and/or 594 nm/619 nm, along with excitation at 405 nm for DAPI. Co-cultured SIMS and NIH 3T3 fibroblasts in alginate hydrogel microtubes were used as the negative control following the immunocytochemistry procedure without addition of primary antibodies (Figure S2).

### 2.10. CellTracker™ Labeling of Mesenchymal and Epithelial Cells

NIH 3T3 and SIMS cells were labeled with CellTracker™ Green CMFDA (C2925) and CellTracker™ Red CMTPX (C34552) from Thermo Fisher Scientific, respectively, with working solutions of 1 µM in serum-free medium (high glucose DMEM and 1% Pen/Strep). CellTracker™ working solution was pre-warmed to 37 °C and incubated with cells for 45 min in a 5% CO_2_ incubator. After incubation, working solution was removed and standard culture medium was added. CellTracker™ Red-labeled SIMS cells and CellTracker™ Green-labeled NIH 3T3 fibroblasts were mixed at a ratio of 1:5 and injected into microtubes, as described previously. Cellular organization images were captured on days 0, 1, and 4 under a Leica Confocal Microscope TCS SP-5 controlled by LAS-AF software using a 10x objective or 63x oil immersion objective (Leica Microsystems, Mannheim, Germany), with excitation/emission of 488 nm/519 nm to track NIH 3T3 fibroblasts in green fluorescence and 594 nm/619 nm to track SIMS epithelial cells in red fluorescence, respectively.

### 2.11. Image Quantification

ImageJ color threshold analysis for quantifying fluorescence intensity was performed by maximum intensity projection over an entire Z-stack of confocal images. Briefly, the fluorescence intensity was extracted from confocal images of anti-EpCAM, anti-vimentin, and DAPI-stained spheroids containing SIMS and NIH 3T3 fibroblasts that were cultured in hydrogel microtubes at differing epithelial to mesenchymal cell ratios (5:1, 5:5, 1:5, 0:5, and 1:0). EpCAM to vimentin ratio was calculated by dividing the fluorescence intensity of EpCAM by that of vimentin using three sets of confocal images. Similarly, EpCAM to DAPI ratio and vimentin to DAPI ratio were obtained. The size of cell clusters of SIMS cells co-cultured with primary E16 mesenchyme cells in hydrogel microtubes on days 1, 4 and 7 in the absence and presence of CaCl_2_ was evaluated by measuring the diameter of 19 or more cell clusters in confocal images for each condition. 

### 2.12. Statistical Analysis

Data were expressed as mean ± standard deviation and analyzed by one-way analysis of variance (ANOVA) with post hoc Tukey HSD (Honestly Significant Difference) test. *p* < 0.05 was considered significant. Each experiment was repeated two to six times. Statistical analysis is provided for experiments with *n* ≥ 3.

## 3. Results

### 3.1. Reproducible Fabrication of Alginate Hydrogel Microtubes

**Figure 1:**
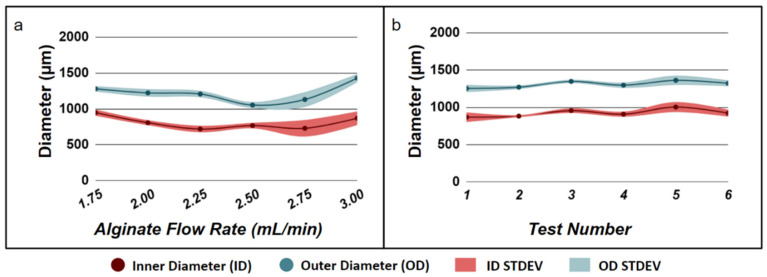
Fabrication of alginate hydrogel microtubes. (**a**) Effect of alginate flow rate on microtube diameter used to determine the optimal alginate flow rate that gives rise to the minimum standard derivation (STDEV) of both the inner diameter and outer diameter of alginate hydrogel microtubes. (**b**) Reproducibility of fabricating hydrogel microtubes at the optimal alginate flow rate 2.00 mL/min.

To test the feasibility of using alginate hydrogel microtubes to facilitate 3D cellular organization of salivary gland epithelial and mesenchymal cells, we chose to fabricate hydrogel microtubes with an inner diameter larger than 500 µm, which allows formation of larger cell clusters and organoids and a wall thickness of ~200 µm, which is within the diffusion limit of oxygen and allows cells to have access to sufficient nutrients [40]. We fabricated alginate hydrogel microtubes in a sterile manner by flowing autoclaved 6% sodium alginate (as the sheath fluid) and 100 mM CaCl_2_ (as the core fluid) through the needle-in-needle device (Device 17–22) into a 50 mL sterile centrifuge tube filled with 100 mM CaCl_2_ [34]. The alginate flow rate was varied from 1.75 mL/min to 3.00 mL/min to determine the effects of the flow rate on the inner diameter of hydrogel microtubes (Figure 1a). Although the alginate flow rates of 1.75 mL/min, 2.00 mL/min, 2.25 mL/min, and 2.50 mL/min generated a minimal level of variation of hydrogel microtubes, flow rates of 1.75 mL/min and 2.50 mL/min produced microtubes with thinner walls, which may make microtubes difficult to handle, while the flow rate of 2.25 mL/min produced microtubes with wall thickness larger than the desired diffusion limit. Therefore, we chose the alginate flow rate of 2.00 mL/min, which generated a minimal level of variation of the inner diameter of hydrogel microtubes as the optimal alginate flow rate and, in particular, produced microtubes with a wall thickness around 200 µm. We then confirmed the reproducibility and repeatability of generating alginate hydrogel microtubes under this optimal alginate flow rate of 2.00 mL/min (Figure 1b). We were able to reproducibly fabricate alginate hydrogel microtubes with an inner diameter of 900 µm and a wall thickness of 190 µm for the co-culturing of salivary gland epithelial and mesenchymal cells.

### 3.2. Effects of Epithelial to Mesenchymal Cell Ratio on Salivary Gland Cell Organization in Pre-Formed Alginate Hydrogel Microtubes

To test the feasibility of co-culturing salivary gland epithelial and mesenchymal cells in pre-formed alginate hydrogel microtubes, we injected the hydrogel microtubes with a mixture of salivary ductal epithelial cells (SIMS) and embryonic fibroblast cells (NIH 3T3) and cultured them in the absence and presence of 25 mM CaCl_2_ (Figure S3). Cell cluster self-organization began as early as day 1, but with undesirable fusion of clusters occurring by day 4 and cell release by day 7, due to a lack of integrity of the hydrogel in the absence of CaCl_2_. The presence of 25 mM CaCl_2_ increased the stability of the hydrogel microtubes and prevented both the unwanted fusion of cell clusters and cell release from the hydrogel microtube on day 7 (compare Figure S3 top and bottom rows). Therefore, 25 mM CaCl_2_ was included in the medium for all subsequent SIMS and NIH 3T3 cells co-culture experiments in hydrogel microtubes.

**Figure 2:**
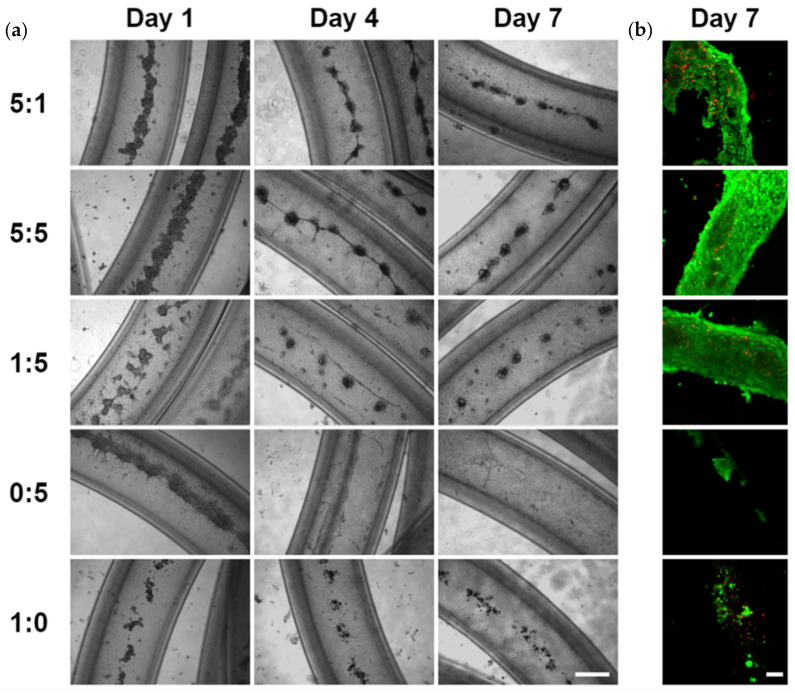
The effect of epithelial to mesenchymal cell ratio on cells co-cultured in alginate hydrogel microtubes for 7 days. SIMS to NIH 3T3 cell ratio of 5:1, 5:5, 1:5. 0:5, and 1:0, respectively. (**a**) Optical images. Scale bar: 500 µm. (**b**) Confocal images of Live/Dead stained cells in microtubes on day 7. Green: live cells. Red: dead cells. Scale bar: 100 µm.

We further determined the most effective epithelial to mesenchymal cell ratio for the organization of cell clusters in hydrogel microtubes using five different SIMS to NIH 3T3 cell ratios, 5:1, 5:5, 1:5, 0:5 (NIH 3T3 fibroblasts only), and 1:0 (SIMS cells only), respectively. The organization of cell clusters, beginning as early as day 1, was confirmed (Figure 2). As shown in Figure 2, cells cultured in a 5:1 ratio did not form individual clusters, and this ratio was determined to be the least effective ratio in forming cell clusters. In contrast, well-defined cell clusters were seen for the 5:5 and 1:5 SIMS:NIH 3T3 ratios by day 7, and cell clusters became more individualized and separated for the 1:5 SIMS:NIH 3T3 ratio. For cells cultured in the µ-Slide 8-Well chamber, the medium was aspirated until 100 µL of medium remained, then 6 µL of Live and Dead Cell Assay stain solution (ab115347, Abcam, Branford, CT, USA) was added to the medium in each well. Additionally, high ratios of epithelial to mesenchymal cells (5:5 or 5:1) gave rise to beads-on-string-like structures. Neither NIH 3T3 fibroblasts alone (0:5) nor SIMS cells alone (1:0) formed individual cell clusters. These results show that the epithelial to mesenchymal cell ratio of 1:5 (SIMS:NIH 3T3) facilitated the formation of separated individual cell clusters.

**Figure 3:**
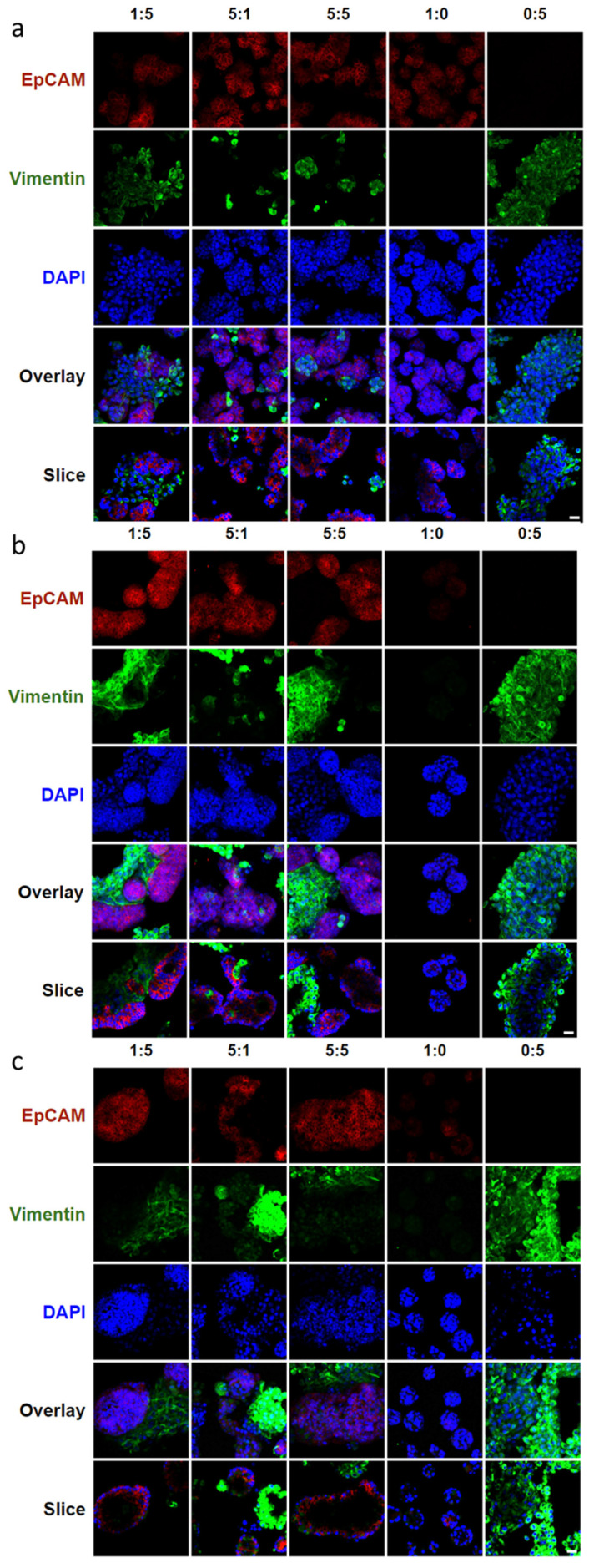
Confocal images showing the effect of epithelial to mesenchymal ratio on marker expression and cellular organization of co-cultured SIMS cells and NIH 3T3 fibroblasts in alginate hydrogel microtubes. (**a**) Day 1. (**b**) Day 4. (**c**) Day 7. Expression of epithelial marker EpCAM in red and mesenchymal marker vimentin in green, co-stained with DAPI in blue. SIMS to NIH 3T3 cell ratio of 5:1, 5:5, 1:5, 0:5, and 1:0 from left to right. Overlay, merged images of EpCAM, vimentin, and DAPI. Slice, cross-sectional view of overlay. Scale bar = 25 µm.

To reveal the cellular reorganization when mesenchymal NIH 3T3 fibroblasts were co-cultured with epithelial SIMS cells in hydrogel microtubes at cell ratios of 5:1, 5:5, and 1:5 over a period of 7 days, cells were immunostained to detect the mesenchyme and the epithelium using the intermediate filament protein, vimentin, and the epithelial marker, EpCAM, respectively. Vimentin is abundant in NIH 3T3 fibroblasts and primary E16 mesenchyme cells and can also be expressed by epithelial cells following an epithelial to mesenchymal transition (EMT). By immunostaining hydrogel-microtube co-cultured epithelial SIMS, a ductal epithelial cell line that should express high levels of EpCAM (red) and mesenchymal NIH 3T3 fibroblasts that express vimentin (green) together with DAPI staining (blue), we could see distinct patterns of cell organization occurring at cell ratios of 5:1, 5:5, and 1:5 (Figure 3). Cells grown at all three ratios exhibited signs of cellular reorganization into spheroidal structures that were partially cavitated by day 7, as seen from individual cross-sectional confocal images captured from the center of these spheroids (Figure 3, bottom row). The DAPI staining in the slices shows a 3D spherical monolayer of cells formed with an absence of cells in the middle. 

The epithelial to mesenchymal cell ratio of 1:5 showed the most uniform cavitated structures throughout the hydrogel microtube. Immunostaining for EpCAM and vimentin confirmed that co-cultured epithelial and mesenchymal cells can organize into cell clusters with cavitation. In particular, the co-cultured epithelial to mesenchymal ratio of 1:5 in hydrogel microtubes exhibited intermingled cell aggregation on day 1 (Figure 3a, slice view of 1:5), EpCAM-expressing epithelial cell clustering on day 4 (Figure 3b, single slice view of 1:5), and cavitation forming a spherical EpCAM-expressing epithelial layer structure on day 7 (Figure 3c, single slice view of 1:5). Compared to epithelial to mesenchymal ratios of 5:1 and 5:5, a ratio of 1:5 gave rise to more uniform EpCAM-expressing epithelial cell clusters (Figure 3). Additionally, these studies showed that SIMS cells alone (1:0) in hydrogel microtubes could only form small cell aggregates with a loss of EpCAM expression by day 7 (Figure 3c, column 1:0). NIH 3T3 fibroblasts alone in hydrogel microtubes could form a vimentin-expressing, fiber-like structure on day 7 (Figure 3c, column 0:5).

**Figure 4:**
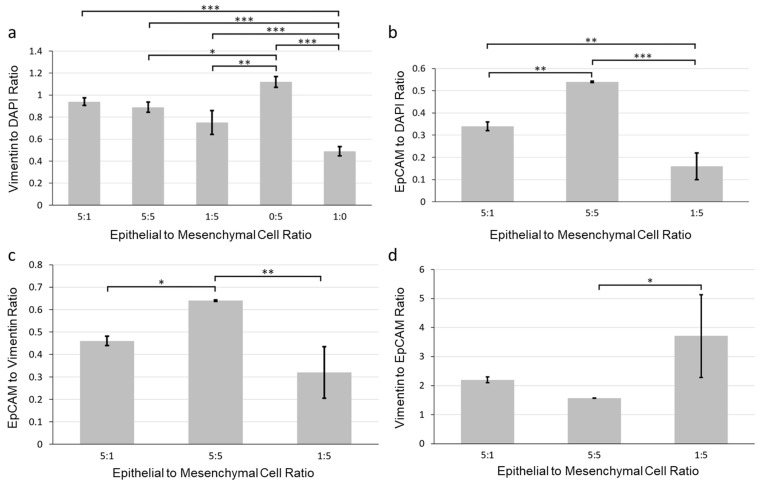
Quantification of the effect of epithelial to mesenchymal cell ratio on marker expression of co-cultured SIMS and NIH 3T3 fibroblasts in alginate hydrogel microtubes for 7 days. (**a**) Vimentin to DAPI ratio. (**b**) EpCAM to DAPI ratio. (**c**) EpCAM to vimentin ratio. (**d**) Vimentin to EpCAM ratio. Epithelial to mesenchymal cell ratios: 5:1, 5:5, 1:5, 0:5 (NIH 3T3 only), and 1:0 (SIMS only). * *p* < 0.05. **, *p* < 0.005. ***, *p* < 0.0001.

To identify the optimal epithelial to mesenchymal ratio, we more closely examined the organization of the cells in the aggregates. We quantified the expression of vimentin and EpCAM from immunostained images of SIMS cells and NIH 3T3 fibroblasts co-cultured for 7 days in alginate hydrogel microtubes at ratios of 5:1, 5:5, and 1:5 (SIMS:NIH 3T3). We examined the vimentin to DAPI intensity ratios (Figure 4a) and EpCAM to DAPI intensity ratios (Figure 4b) and further compared the EpCAM to vimentin intensity ratio (Figure 4c). We found that salivary gland epithelial SIMS cells alone cultured in hydrogel microtubes (1:0) expressed vimentin (Figure 4a) on day 7, suggesting that these epithelial cells might undergo an EMT in the absence of mesenchymal cells. The expression of vimentin was not lower in the 5:1 ratio culture that had four-fold fewer mesenchymal cells than in the 5:5 ratio, indicating that the epithelial cells might also undergo EMT in the 5:1 ratio condition (Figure 4a). Cells in the 5:5 ratio culture showed the highest EpCAM expression (Figure 4b). However, since there were five times fewer initially seeded epithelial cells in the 1:5 than 5:5 ratio cultures, the EpCAM per epithelial cell would be the highest at the 1:5 culture. There was no significant difference in the EpCAM to vimentin ratio between the 1:5 and 5:1 cultures (Figure 4c). We also determined that the epithelial to mesenchymal ratio of 1:5 showed the highest vimentin to EpCAM ratio (Figure 4d), indicating the retention of stromal cells during co-culture. In addition, the epithelial to mesenchymal ratio of 1:5 gave rise to individual cell clusters (Figure 2a), resulting in uniform spheroids (Figure 3c). Therefore, for all further experimental procedures, we chose an epithelial to mesenchymal cell ratio of 1:5, which supported uniform epithelial cell cluster organization with cavitation. 

### 3.3. Facilitation of 3D Salivary Gland Cell Organization by Co-Culture of Epithelial SIMS Cells and NIH 3T3 Fibroblasts in Alginate Hydrogel Microtubes 

**Figure 5:**
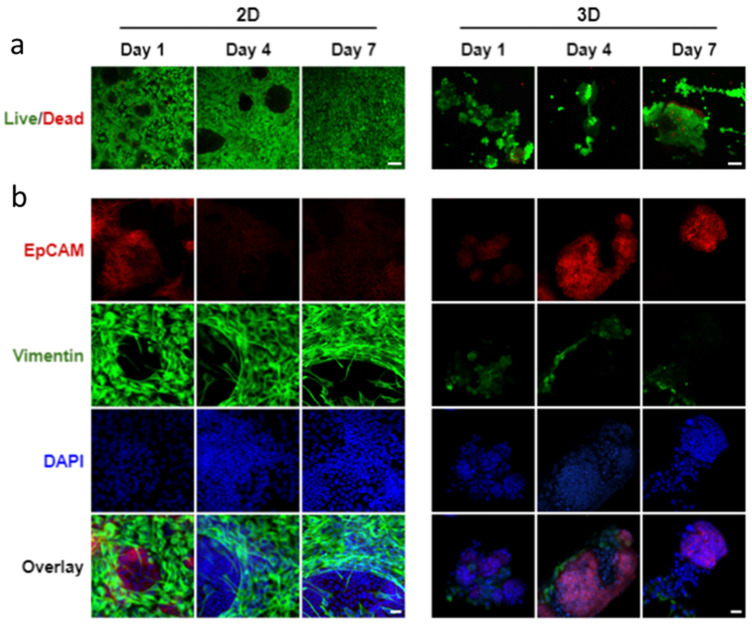
Confocal images of co-cultured SIMS cells and NIH 3T3 fibroblasts at a cell ratio of 1:5 in 2D chamber slides vs. 3D alginate hydrogel microtubes supplemented with 25 mM CaCl_2_ for 7 days. (**a**) Live/Dead Cell Assay to stain live cells in green and dead cells in red. Scale bar = 100 µm. (**b**) Expression of epithelial marker, EpCAM (red) and mesenchymal marker, vimentin (green) co-stained with DAPI (blue). Overlay, merged images of EpCAM, vimentin, and DAPI. Scale bar = 25 µm.

After determining the most effective epithelial to mesenchymal cell ratio as 1:5, we compared cell organization of co-cultured SIMS cells and NIH 3T3 fibroblasts using alginate hydrogel microtubes as a 3D environment with 2D cultures on chamber slides (Figure S4 and Figure 5). While the 2D culture showed cell monolayer morphology, and while the 3D culture exhibited cell aggregation, both 2D and 3D cell culturing environments showed that the majority of cells are viable on days 1, 4, and 7 (Figure 5a). Noticeable 3D cell aggregate structures formed when co-culturing SIMS cells and NIH 3T3 fibroblasts in the hydrogel microtube (Figure 5a, right panel), indicating a strong self-organizing characteristic between the 3D co-cultured epithelial and mesenchymal cell types.

We further compared the epithelial cell marker expression in co-cultured SIMS and NIH 3T3 between the 2D cell culture on the chamber slides and the 3D culture in hydrogel microtubes. When co-cultured in 2D, a 2D monolayer structure of cells formed, in which EpCAM-expressing epithelial SIMS cells were outcompeted by the growth rate of vimentin-expressing NIH 3T3 fibroblasts (Figure 5b, left panel). When co-cultured in 3D hydrogel microtubes, EpCAM was highly expressed on days 4 and 7 (Figure 5b, right panel), with the EpCAM-expressing cells organized into 3D spheroidal monolayer cell clusters (Figure S5).

**Figure 6:**
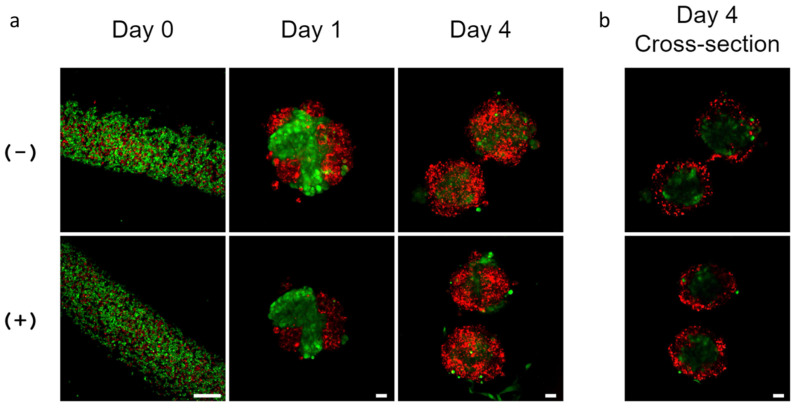
Cellular organization of co-cultured SIMS cells and NIH 3T3 fibroblasts in alginate hydrogel microtubes. (**a**) Confocal images of CellTracker™ Red CMTPX-labeled SIMS cells and CellTracker™ Green CMFDA-labeled NIH 3T3 fibroblasts co-cultured in microtubes for 4 days. Scale bar = 250 µm. (**b**) Confocal images of the cross-sectional view of cell clusters formed by co-culture of SIMS and NIH 3T3 in microtubes for 4 days. (−) Without and (+) with 25 mM CaCl_2_. Scale bar = 25 µm. Red: SIMS cells. Green: NIH 3T3 cells.

To monitor cellular organization in hydrogel microtubes over time, we labeled SIMS cells with CellTracker™ Red CMTPX and NIH3T3 fibroblasts with CellTracker™ Green CMFDA. These cells were injected into alginate hydrogel microtubes and co-cultured for 4 days in the absence or presence of 25 mM CaCl_2_ (Figure 6a). Immediately after injection, live imaging of CellTracker™ Red-labeled SIMS cells and CellTracker™ Green-labeled NIH 3T3 fibroblasts showed that SIMS cells and fibroblasts were mixed and distributed uniformly in the hydrogel microtube. On day 1, these cells aggregated in hydrogel microtubes, forming intermingled spheroidal cell clusters (Figure 6a). On day 4, CellTracker™ Red-labeled SIMS cells were localized on the surface of the cell cluster, while the green-labeled NIH 3T3 cells appeared to be inside the cluster (Figure 6a). The cross-sectional view of the co-culture in the microtubes demonstrated that CellTracker™ Red-labeled epithelial cell clusters exhibited cavitation (Figure 6b), confirming epithelial cell organization supported by mesenchymal cells in alginate hydrogel microtubes.

### 3.4. Co-Culture of Salivary Gland Epithelial SIMS Cells and Primary E16 Mesenchyme Cells in Hydrogel Microtubes

**Figure 7:**
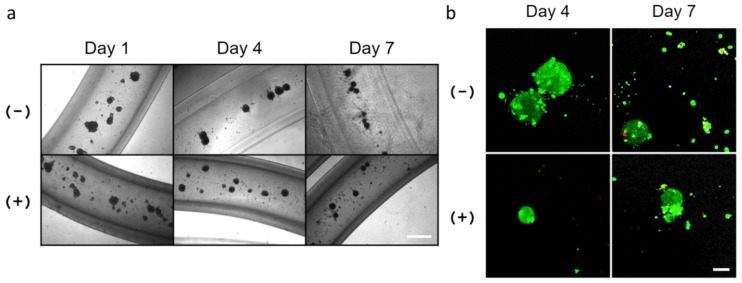
Co-culture of SIMS cells and primary E16 salivary mesenchyme cells at a cell ratio of 1:5 in alginate hydrogel microtubes for 7 days. (**a**) Optical images of co-cultured SIMS and E16 cells in microtubes. Scale bar = 500 µm. (**b**) Confocal images of Live/Dead Cell Assay (live cells in green and dead cells in red) of co-cultured SIMS and E16 cells in microtubes. Scale bar = 100 µm. (−) Without and (+) with 25 mM CaCl_2_.

To determine if primary salivary mesenchyme would also support cellular organization of SIMS cells, mouse primary E16 mesenchyme cells were isolated from embryonic salivary gland stroma and co-cultured with SIMS cells at a 1:5 ratio (SIMS:E16) in alginate hydrogel microtubes (Figure 7). On day 1, we observed multiple cell clusters formed in hydrogel microtubes; continued culturing through days 4 and day 7 revealed additional modification of these cell clusters by compaction into tighter clusters (Figure 7a), the majority of which remained viable (Figure 7b). 

**Figure 8:**
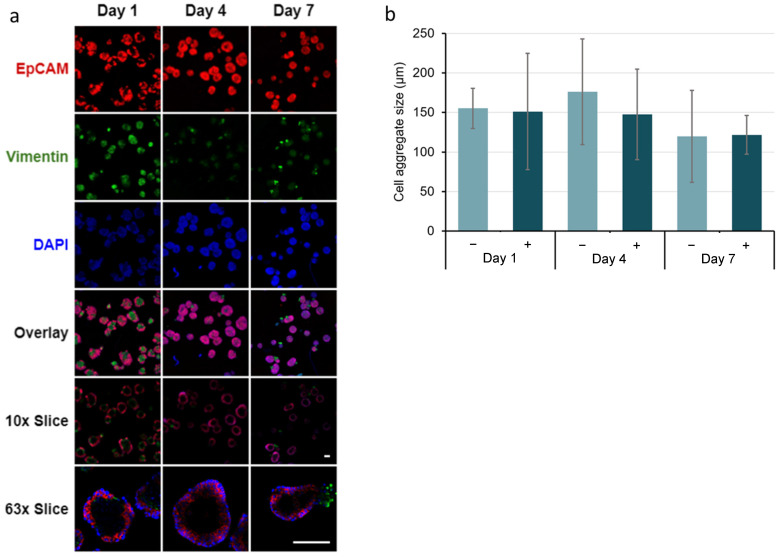
Immunocytochemistry analysis of co-cultured SIMS and E16 salivary mesenchyme cells at a cell ratio of 1:5 in alginate hydrogel microtubes supplemented with 25 mM CaCl_2_ for 7 days. (**a**) Confocal images of epithelial marker (EpCAM, red) and mesenchymal marker (vimentin, green) expression co-stained with DAPI (blue). Overlay, merged images of EpCAM, vimentin, and DAPI. 10x Slice, cross-sectional view of the center with 10x objective lens. 63x Slice, cross-sectional view of the center with 63x oil immersion lens. Scale bar = 100 µm. (**b**) Quantification of the size of the salivary spheroids formed from co-cultured SIMS and E16 cells in hydrogel microtubes in absence (−) and presence (+) of 25 mM CaCl_2_. *p* > 0.05, not significant.

Immunocytochemistry of co-cultured salivary gland epithelial SIMS and E16 mesenchyme cells in hydrogel microtubes showed that the EpCAM expression was maintained throughout the culture (Figure 8a and Figure S6). In contrast, vimentin expression was initially observed on day 1, decreased by day 4, and began to increase again by day 7. The single slice/cross-sectional view of co-cultured SIMS cells showed the formation of the spheroidal cell clusters (Figure 8a, rows 10x Slice or 63x Slice).

Additionally, we also demonstrated that after culturing in hydrogel microtubes for 7 days, primary E16 mesenchyme cells released from the microtubes could be re-plated in a 6-well plate and proliferate for 7 days (Figure S7a), maintaining the expression of the mesenchymal marker, vimentin (Figure S7b), indicating that cells grown in hydrogel microtubes retained their proliferation potential.

## 4. Discussion

We have demonstrated that salivary gland epithelial and mesenchymal cells can be injected and co-cultured in pre-formed alginate hydrogel microtubes. These hydrogel microtubes allow dynamic cell–cell interaction, which has the potential to mimic highly dynamic epithelial cell movement observed in epithelial buds during the developmental process of branching morphogenesis [41]. Both NIH 3T3 fibroblasts and primary E16 salivary mesenchyme cells support SIMS salivary gland epithelial cell organization into 3D cavitated structures in these hydrogel microtubes. Alginate hydrogel microtubes provide a 3D culture microenvironment for sustaining cellular organization to cell aggregation and cavitation, necessary steps toward designing an engineered salivary gland tissue structure [8].

### 4.1. Pre-Formed Alginate Hydrogel Microtubes for Cell Injection and Cellular Organization

Alginate hydrogel microtubes have been previously used for stem cell expansion and differentiation [30,31,32,33]. Using our needle-in-needle, device-based method, we can not only encapsulate cells in hydrogel microtubes, but also fabricate pre-formed hydrogel microtubes as an “off-the-shelf” 3D culture system that allows injection of cell types of interest at any cell seeding density and cell ratio for co-culture or multi-culture. In particular, by using alginate hydrogel microtubes, the hollow core offers a non-restricted space, allowing cell migration and aggregation and providing access to nutrients in the immediate vicinity of cell clusters [42,43,44]. The use of 6% sodium alginate makes these hydrogel microtubes strong enough to be handled for cell injection and long-term cell culture compared to conventional hydrogel microtubes made of low viscosity sodium alginate [45] or low concentration (e.g., 1–2%) sodium alginate [31,32,33]. Supplementing culture media with 25 mM CaCl_2_ further improved the stability of these hydrogel microtubes by maintaining structural integrity during culture. Having analyzed the effects of calcium addition in each experiment we performed, there was no adverse effect of the additional 25 mM CaCl_2_ on cell viability and cellular organization. For example, both co-cultured SIMS/NIH 3T3 and co-cultured SIMS/E16 mesenchyme cells in hydrogel microtubes exhibited a similar cellular organization (e.g., cell migration, aggregation, and epithelial cavitation) in the presence of 25 mM CaCl_2_ to those in its absence (compare Figure 5 to Figure S4 and Figure 8 to Figure S6). Our results show that the 3D co-culture of SIMS and NIH 3T3 cells in hydrogel microtubes maintained EpCAM expression and facilitated EpCAM-expressing epithelial spheroid formation, while the 2D co-culture exhibited high vimentin expression along with the loss of EpCAM expression on day 7 (Figure 5b). These results highlight the importance of the 3D co-culture for cellular organization. Since salivary gland ductal cells are sensitive to extracellular [Ca^2+^] [46], further study is warranted to determine the minimum CaCl_2_ concentration needed to ensure the stability of alginate microtubes during long-term cell culture and to maintain normal salivary gland function. Since the calcium concentration in saliva is in the range of 1.2 mM–2.8 mM [47], in future studies, we will determine if 1 mM–2 mM CaCl_2_ medium supplementation is sufficient to stabilize the alginate hydrogel microtube during culture. We will also examine the feasibility of using Ba^2^+ to stabilize the alginate hydrogel microtube since Ba^2^+ yields the most stable alginate hydrogel relative to other divalent cations [48].

### 4.2. 3D Co-Culture of Salivary Gland Epithelial and Mesenchymal Cells for Cavitation in Alginate Hydrogel Microtubes 

Alginate hydrogel microtubes provide a 3D microenvironment for dynamic salivary gland epithelial and mesenchymal cell interaction and self-organization into 3D cavitated structures. Co-culture models have used the Transwell^®^ inserts by culturing primary salivary gland cells from mice [11], rats [12,13], or humans [14] on the polyester or polycarbonate membrane and culturing mesenchymal stem cells in the lower chamber. In these experiments, MSCs adopted a salivary gland epithelial phenotype, suggesting mesenchymal to epithelial transition. For example, when co-cultured with human salivary gland biopsies, human MSCs demonstrated a salivary epithelial phenotype, including tight junction structures and numerous secretory granules, expression of tight junction proteins (e.g., claudins, occludin, junctional adhesion molecule-A, and ZO-1) as well as other epithelial markers (e.g., aquaporin-5, α-amylase (α-AMY), and E-cadherin), and exhibited a high transepithelial electrical resistance [14]. When co-cultured with mouse salivary gland cells, bone marrow-derived MSCs underwent morphological changes and expressed salivary acinar markers and ductal markers at the protein and mRNA levels that resembled salivary gland cells [11]. These Transwell^®^ chamber-based co-culture studies only allow indirect epithelial cell–mesenchymal cell interaction. However, indirect epithelial–mesenchymal cell interaction only induced 2D branching morphogenesis, while direct epithelial–mesenchymal cell interaction was needed to facilitate 3D branching [15]. Our co-culture in alginate hydrogel microtubes provides a new avenue for studying not only indirect but also direct epithelial and mesenchymal cell interactions in 3D. Since these hydrogel microtubes are transparent, cell–cell interactions and cellular organization can be monitored under an inverted optical microscope on daily basis. 

The mesenchymal component of salivary glands is of paramount importance for the induction of epithelial differentiation and branching morphogenesis during salivary gland tissue development [5,14,49,50,51,52]. Primary or established epithelial salivary gland cells cultured alone grew very slowly and were difficult to culture [53]. Our results show that epithelial salivary gland SIMS cells cultured alone in hydrogel microtubes showed only small cell aggregates (Figure 2, row 1:0), and loss of EpCAM expression occurred on day 4 and day 7 (Figure 3, row 1:0). EpCAM is a relatively weak cell–cell adhesion molecule that can modify cell–cell contact adhesion strength and tissue plasticity [54]. It is also an epithelial marker of both acinar and ductal epithelial cells, with acinar salivary gland cells showing relatively low EpCAM expression [55,56] and ductal salivary gland cells showing high expression [8]. The salivary epithelial model cell type that we used, SIMS, is a ductal epithelial cell line and, therefore, expresses high levels of EpCAM. For the 3D co-culture of SIMS with NIH 3T3 fibroblasts (Figure S5) or E16 mesenchyme cells (Figure 8a, row of 63x Slice) at an epithelial to mesenchymal cell ratio of 1:5, both gave rise to similar epithelial cavitated structures that retained EpCAM expression, highlighting the importance of the mesenchymal component for salivary gland tissue morphogenesis, consistent with our past work [9]. 

A variety of epithelial to mesenchymal cell ratios have been evaluated in the above-mentioned Transwell^®^ chamber-based, indirect co-culture experiments, including 19:1 (mouse SMG cells:induced pluripotent stem cells) [57], 6:1 (rat SGC:MSC) [11], 4:1 (rat acinar cells:MSC) [12,13], 2:1, 1:1, and 1:2 (mouse SMG epithelial cells:MSCs) [16]; and 1:1 (mouse salivary gland cells:human adipocyte-derived MSC) [58]. Our alginate hydrogel microtubes allowed us to evaluate the effects of the epithelial to mesenchymal cell ratio on cellular organization, leading us to identify a 1:5 epithelial to mesenchymal cell ratio as optimal for cell aggregation and cavitation. Cavitation and lumen formation in the ducts is one of the first steps to branching morphogenesis [59]. 

Three-dimensional salivary gland organoid cultures have been formed using hanging drop culture [60], hydrogel encapsulation (e.g., hyaluronic acid) [61,62], and multi-step differentiation of mouse embryonic stem cells in the presence of Matrigel [63]. In addition, 3D cultures in Matrigel matrices have been performed using a variety of salivary gland tissues, including mouse SMG epithelium [64], human parotid and SMG cells [53], single human salivary cells [65], human SMG stem/progenitor cells [66], mouse and human SMG stem/progenitor cell-derived salispheres [67,68,69], co-cultured mouse salivary gland progenitor cells and MSCs [16,17], and salispheres with E16 mesenchyme in the presence of fibroblast growth factors (FGFs), such as FGF2 [38]. These Matrigel-based 3D cultures provide valuable information on branching morphogenesis and serve as in vitro models. However, Matrigel is mouse tumor-derived and poorly defined, which is not ideal for in vivo tissue regeneration. Moreover, cells cultured in Matrigel cannot migrate freely. Our alginate hydrogel microtubes provide an alternative and well-defined 3D culture microenvironment that does not contain adhesive molecules or growth factors, while allowing cells to migrate freely and facilitate cavitation. Additionally, compared to a conventional hanging drop culture and cell aggregation in ultralow adhesion plates or microfabricated cell-repellent wells, our hydrogel microtubes not only facilitate self-assembly of cavitated organoids of homogeneous size and shape, but also prevent unwanted organoid fusion as well as loss of organoids during medium changes.

### 4.3. Future Strategies to Induce Branching Morphogenesis from Cavitated Structures Formed in Alginate Hydrogel Microtubes 

To induce further development of the cavitated tissue aggregates, we included primary mesenchyme cells. The mesenchyme has the capacity to direct bud development through FGF10 signaling [70], and adding SMG mesenchymal cells to epithelial cell culture facilitates branching morphogenesis [64]. Mouse bone marrow-derived MSCs also showed the capacity to support branching of SMG epithelium in 2D and 3D cultures [16]. In particular, the transplantation of salivary gland organoids combined with mouse embryonic salivary gland mesenchyme promoted salivary gland organoid maturation in vivo [66]. For in vitro cavitated salivary gland tissue maturation, we may inject additional primary E16 salivary mesenchyme into alginate hydrogel microtubes containing epithelial cavitated structures from our original co-culture method or release these epithelial cavitated structures from alginate hydrogel microtubes and then co-culture them with additional E16 mesenchyme cells. 

With further manipulation of growth factors and extracellular matrix (ECM) proteins, salivary gland regeneration may be advanced through the use of alginate hydrogel microtubes for salivary gland cell migration, aggregation, lumen formation, and cavitation. In addition to facilitating cell–cell interaction, the alginate hydrogel microtube has the potential for the sustainable delivery of matrix proteins or controlled release of growth factors. For example, FGF proteins can act in concert with ECM proteins, such as fibronectin to reorganize cells during branching morphogenesis [17,41,71] and laminin to induce epithelial cell differentiation and tight junction formation [72,73]. Laminin is a significant component of Matrigel and can substitute for Matrigel in organoid assays [9]. Fibronectin is required for cleft formation in branching morphogenesis, including salivary gland development [74,75]. The supplementation of fibronectin in the presence of FGF7 induced branching of the epithelium and caused ductal elongation within salivary gland cell clusters grown in Matrigel in a fibronectin dose-dependent manner [17]. Laminin-111 on top of a floating filter membrane supported end bud expansion in the presence of FGF7 or induced ductal elongation in the presence of FGF10 [76]. Laminin-111 peptide-modified hydrogel induced the formation of branches when exposed to FGF7 or promoted proliferation when exposed to FGF10 [77]. We envision that using the alginate hydrogel microtubes to co-culture primary or stem cell-derived salivary progenitors and mesenchymal stromal cells in the presence of growth factors or small molecules and ECM proteins that promote secretory acini morphogenesis could generate uniform, reproducible, functional, and cavitated cell clusters for salivary functional restoration. This culture method could lead to an optimized salivary gland organoid culture platform for understanding epithelial–mesenchymal cell interactions, drug testing, and even developing implantable patient-specific organoids for treatment of salivary gland dysfunction. 

## 5. Conclusions

We have established a co-culture of salivary gland epithelial and mesenchymal cells by injecting cell mixtures into pre-formed alginate hydrogel microtubes, which facilitate direct, 3D, and heterotypic cell–cell interactions, and the self-assembly of cavitated epithelial organoids. These microtubes allow monitoring of cellular organization while providing a protective barrier from cell-cluster loss during medium changes. Through use of these hydrogel microtubes for co-culture, we have been able to evaluate the effects of the epithelial to mesenchymal ratio on cellular organization and determine the optimal epithelial to mesenchymal cell ratio for cell reorganization to occur in this context. Co-cultured SIMS salivary gland epithelial cells and NIH 3T3 fibroblasts remained viable and organized into an EpCAM-expressing, cavitated, and epithelial layer of 3D spheroidal cell clusters in hydrogel microtubes after 7 days, whereas a 2D co-culture in cell culture slide chambers only formed cavities among a monolayer of vimentin-expressing mesenchymal cells, while epithelial cells lost EpCAM expression. Co-culturing SIMS cells with primary E16 salivary mesenchyme cells in hydrogel microtubes produced 3D cavitated spheroidal epithelial cell clusters. These data demonstrate that mesenchymal cells support salivary gland epithelial cell organization in hydrogel microtubes. Altogether, alginate hydrogel microtubes provide a co-culture platform for further understanding epithelial and mesenchymal interactions during tissue morphogenesis and for optimizing salivary gland organoid culture for applications in drug screening and in salivary gland tissue regeneration.

## Data Availability

The data presented in this study are available in the article and Supplementary Materials.

## Supplementary Materials

The following are available online at https://www.mdpi.com/article/10.3390/bioengineering9010038/s1. Materials and Methods: Co-culture of Organoids Derived from Epithelial Clusters and Mesenchymal Cells [9]. Figure S1: Photos of alginate hydrogel microtubes. Figure S2: Representative confocal images of negative and positive controls of immunocytochemistry. Figure S3: Optical images of co-cultured SIMS cells and NIH 3T3 fibroblasts (1:5 ratio) in alginate hydrogel microtubes for 7 days. Figure S4: Confocal images of co-cultured SIMS cells and NIH 3T3 fibroblasts at a cell ratio of 1:5 in 2D chamber slides vs. 3D alginate hydrogel microtubes for 7 days in the absence of 25 mM CaCl_2_. Figure S5: Confocal images of the cross-sectional view at the center of co-cultured SIMS cells and NIH 3T3 fibroblasts at a cell ratio of 1:5 in alginate hydrogel microtubes for 7 days. Figure S6: Confocal images of marker expression in co-cultured SIMS and E16 salivary mesenchyme cells at a cell ratio of 1:5 in alginate hydrogel microtubes in the absence of 25 mM CaCl_2_ for 7 days. Figure S7: Images of primary E16 mesenchyme cells re-plated in a 6-well plate showing that these cells could proliferate after culturing in alginate hydrogel microtubes for 7 days followed by cell release from the microtube.

## Abbreviations

2D, two-dimension(al); 3D, three-dimension(al); α-AMY, α-amylase; ANOVA, one-way analysis of variance; BMP-6, Bone Morphogenetic Protein 6; CEBPB, CCAAT Enhancer Binding Protein Beta; DAPI, 4′,6-diamidino-2-phenylindole; E16, embryonic day 16; DMEM, Dulbecco’s Modified Eagle’s Medium; DMEM/F12, Dulbecco’s Modified Eagle Medium: Nutrient Mixture F-12; ECM, extracellular matrix; EMT, epithelial–mesenchymal transition; EpCAM, epithelial cell adhesion molecule; FBS, fetal bovine serum; FGF, fibroblast growth factor; HEPES, (4-(2-hydroxyethyl)-1-piperazineethanesulfonic acid; IACUC, Institutional Animal Care and Use Committee; ID, inner diameter; MET, mesenchymal–epithelial transition; mRNA, messenger ribonucleic acid; MSC, mesenchymal stem cells; OD, outer diameter; PBS, phosphate buffer saline; PFA, paraformaldehyde; PGS, poly (glycerol sebacate); PLGA, poly (lactic co glycolic acid); SIMS, submandibular immortalized mouse ductal salivary gland cell line; SMG, submandibular salivary gland; STDEV, standard deviation; ZO-1, Zonula occludens-1.
