# Supplementary material for: Alginate Hydrogel Microtubes for Salivary Gland Cell Organization and Cavitation
Source: Bioengineering (Basel). 2022 Jan 15;9(1):38. doi: 10.3390/bioengineering9010038 (PMC8773299; doi:10.3390/bioengineering9010038)
Supplement: Supplementary file 1 [file bioengineering-09-00038-s001.zip › bioengineering-1504214-supplementary.pdf]

Supplementary Materials

Materials and Methods

Co-culture of Organoids Derived from Epithelial Clusters and Mesenchymal Cells [9]

Primary E16 mesenchyme cells (derived from 3 glands, approximately 0.5×10^6 cells) were seeded on Nuclepore filters floating on DMEM/F12 with 10% fetal bovine serum (FBS) (Life Technologies) supplemented with 100 U/mL penicillin and 100 mg/mL streptomycin (Life Technologies) for 4 hours. Epithelial clusters (derived from 0.5 glands, approximately 5×10^3 cells in 100 clusters) were plated on top of the mesenchyme feeder layer. Cells were co-cultured for 7 days with a media change on day 4.

Figures S1–S7

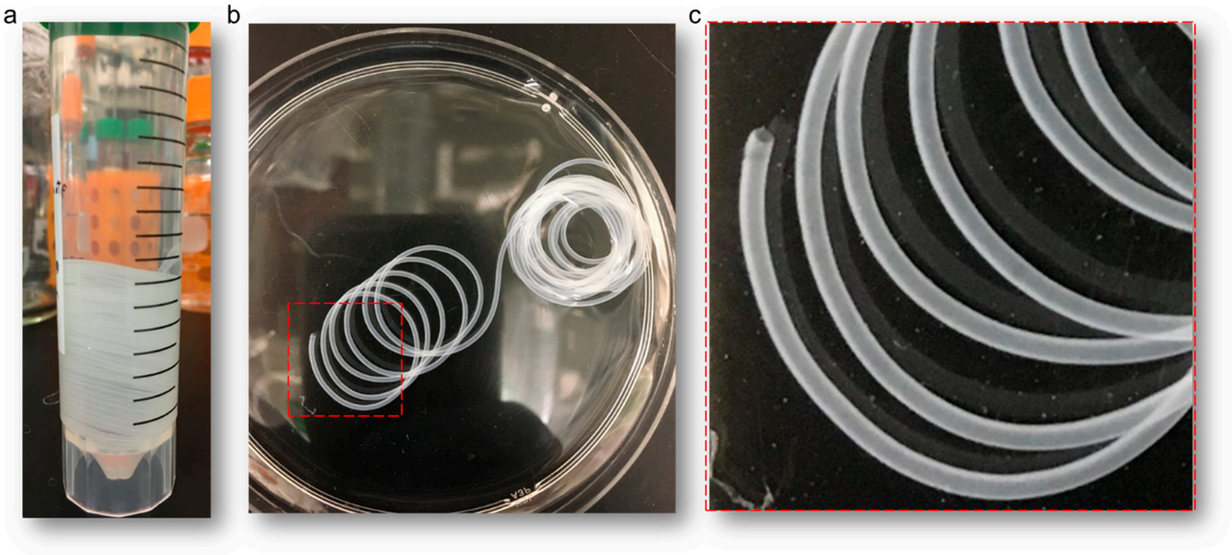

Figure S1. Photos of alginate hydrogel microtubes. (a) Stacks of the alginate microtube stored in a 50-mL centrifuge tube. (b) The alginate microtube in a 100-mm petri dish showing the open end of the microtubes. (c) Enlarged view of the open end of the alginate microtube for cell injection.

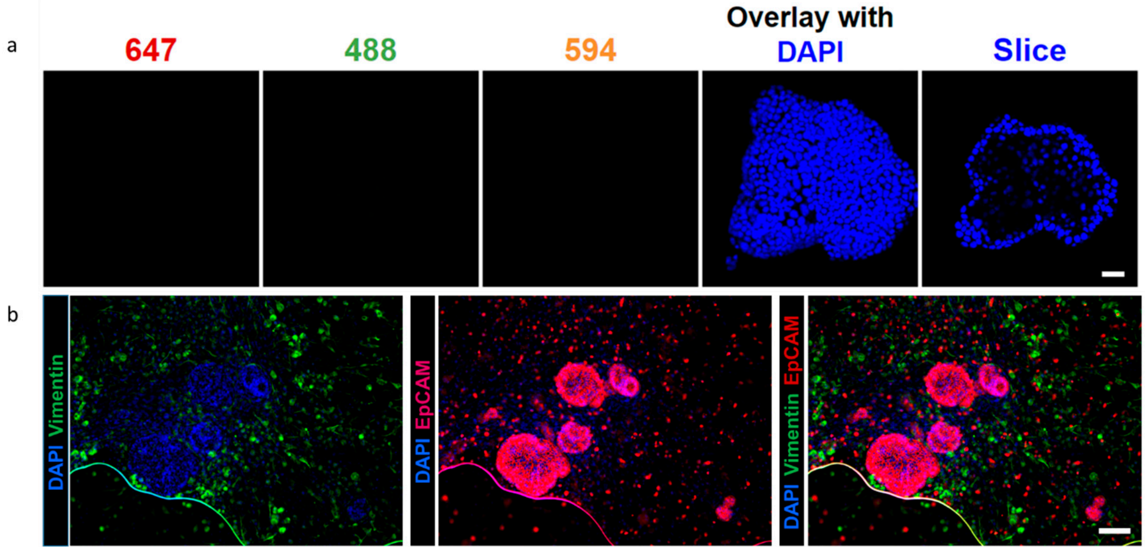

Figure S2. Representative confocal images of negative and positive controls of immunocytochemistry. (a) Co-cultured SIMS cells and NIH 3T3 fibroblasts in alginate hydrogel microtubes following the immunocytochemistry procedure without adding primary antibodies, stained with DAPI. No fluorescence was observed at excitation wavelengths of 647, 488, and 594 nm, serving as negative controls for Alexa Fluor 647-conjugated EpCAM and Alexa Fluor 488-conjugated vimentin. Scale bar = 25 µm. (b) Salivary gland organoids derived from E16 epithelial (EPCAM) and stromal cells (vimentin) cultured for 7 days demonstrating epithelial- and stromal-cell positive immunocytochemistry controls. While vimentin (green) staining is observed in panels 1 and 3, membrane localized EpCAM (magenta) is apparent in panels 2 and 3. Scale bar = 200 µm.

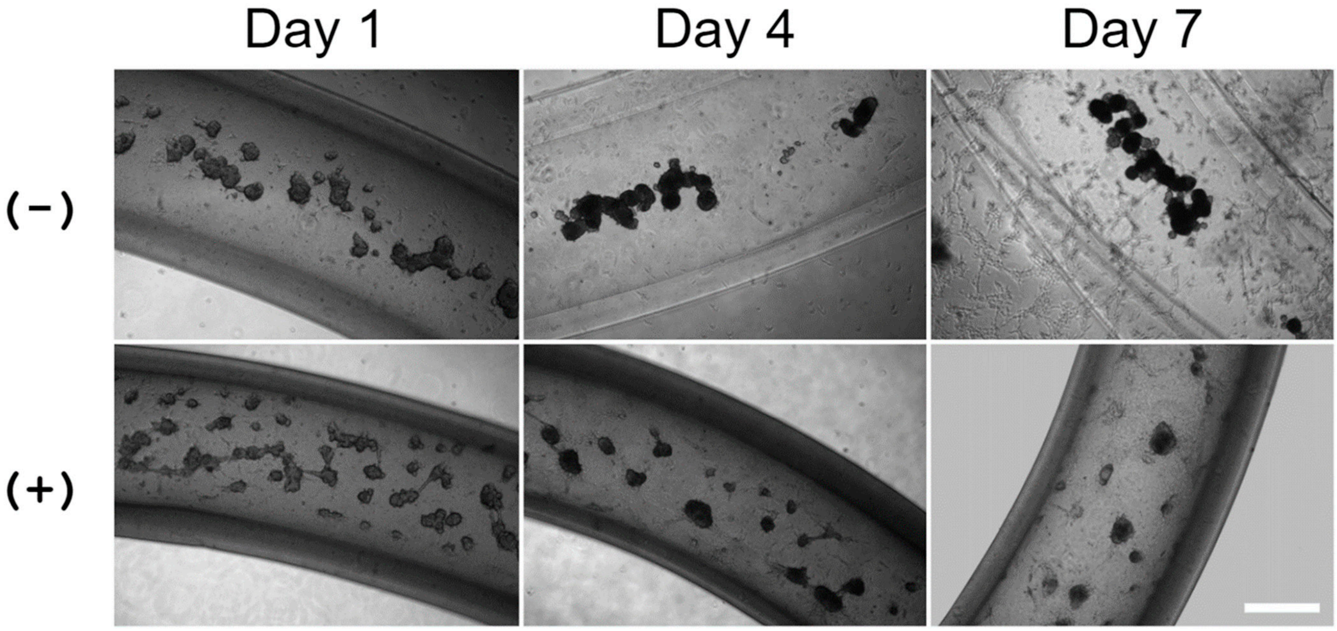

Figure S3. Optical images of co-cultured SIMS cells and NIH 3T3 fibroblasts (1:5 ratio) in alginate hydrogel microtubes for 7 days. (−) Without 25 mM $CaCl_2$ and (+) with 25 mM $CaCl_2$. Scale bar = 500 μm.

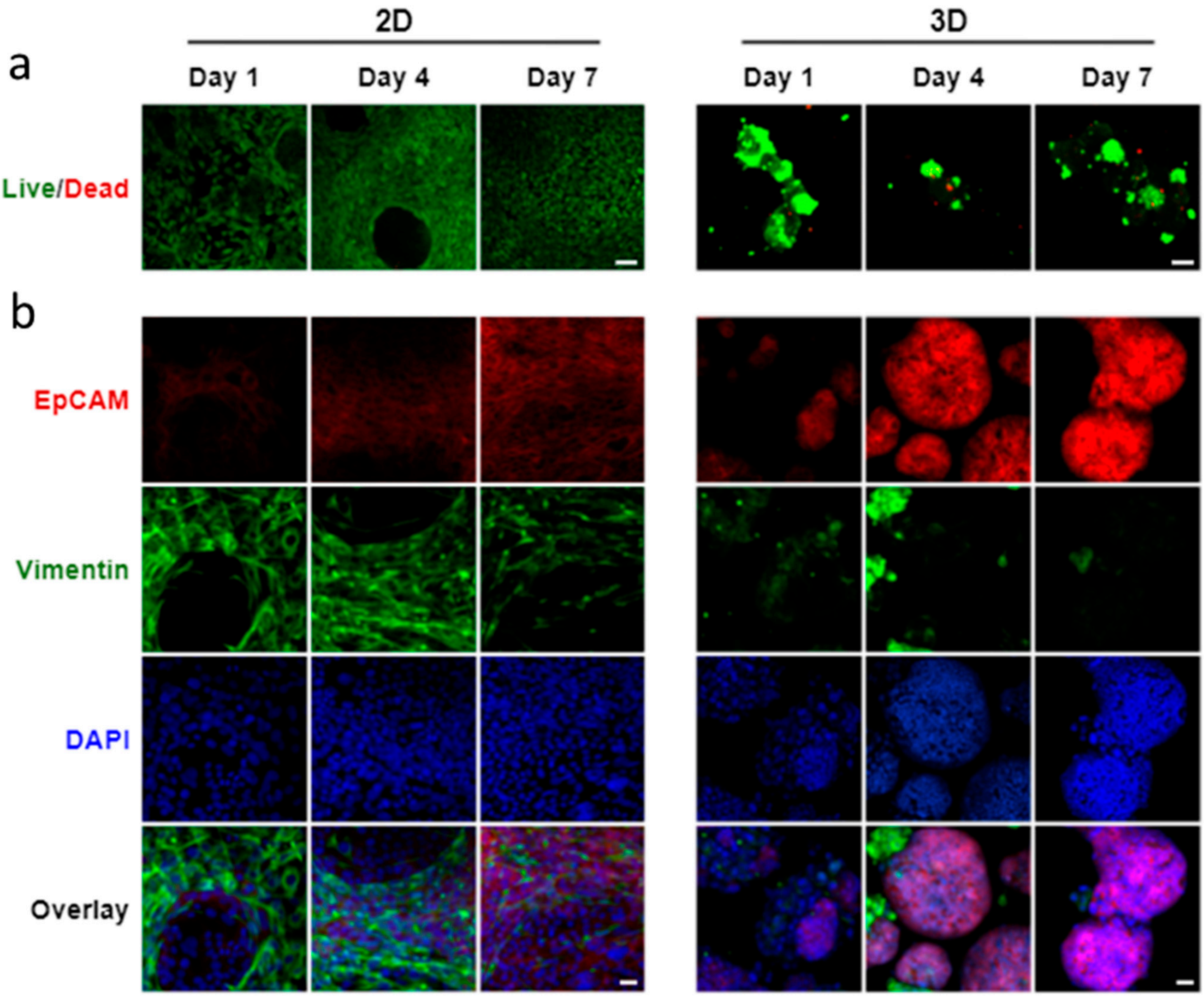

Figure S4. Confocal images of co-cultured SIMS cells and NIH 3T3 fibroblasts at a cell ratio 1:5 in 2D chamber slides vs. 3D alginate hydrogel microtubes for 7 days in the absence of 25 mM $CaCl_2$. (a) Live/Dead Cell Assay to stain live cells in green and dead cells in red. Scale bar = 100 μm. (b) Expression of epithelial marker, EpCAM (red) and mesenchymal marker, vimentin (green) co-stained with DAPI (blue). Overlay, merged images of EpCAM, vimentin, and DAPI. Scale bar = 25 μm.

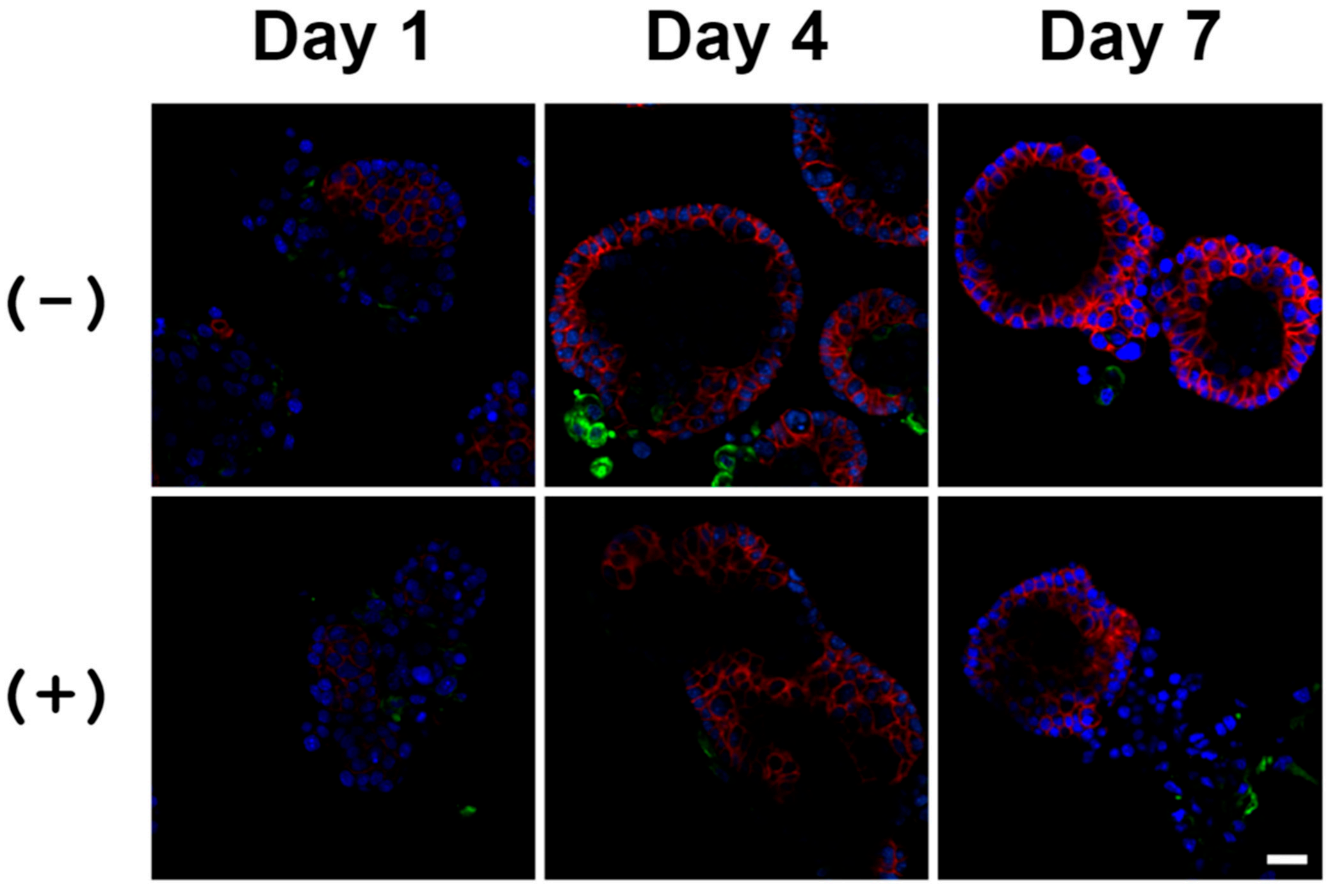

Figure S5. Confocal images of the cross-sectional view at the center of co-cultured SIMS cells and NIH 3T3 fibroblasts at a cell ratio 1:5 in alginate hydrogel microtubes for 7 days. (−) without 25 mM $CaCl_2$ and (+) with 25 mM $CaCl_2$. Observation under 63x oil immersion lens showed expression of epithelial marker EpCAM (red) and mesenchymal marker vimentin (green) co-stained with DAPI (blue). Scale bar = 25 μm.

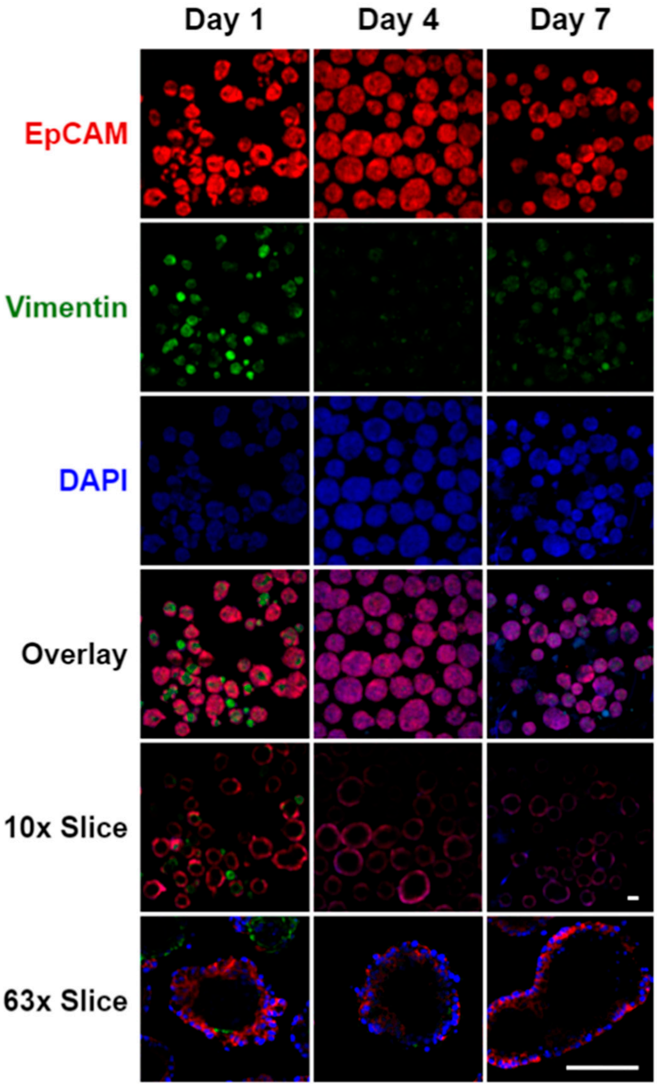

Figure S6. Confocal images of marker expression in co-cultured SIMS and E16 salivary mesenchyme cells at a cell ratio of 1:5 in alginate hydrogel microtubes in the absence of 25 mM $CaCl_2$ for 7 days. Red, epithelial marker EpCAM. Green, mesenchymal marker vimentin. Blue, DAPI stained nuclei. Overlay, merged images of EpCAM, vimentin, and DAPI. 10x Slice, cross-sectional view of the center with 10x objective lens. 63x single confocal slice, cross-sectional view of the spheroid center with 63x oil immersion lens. Scale bar = 100 µm.

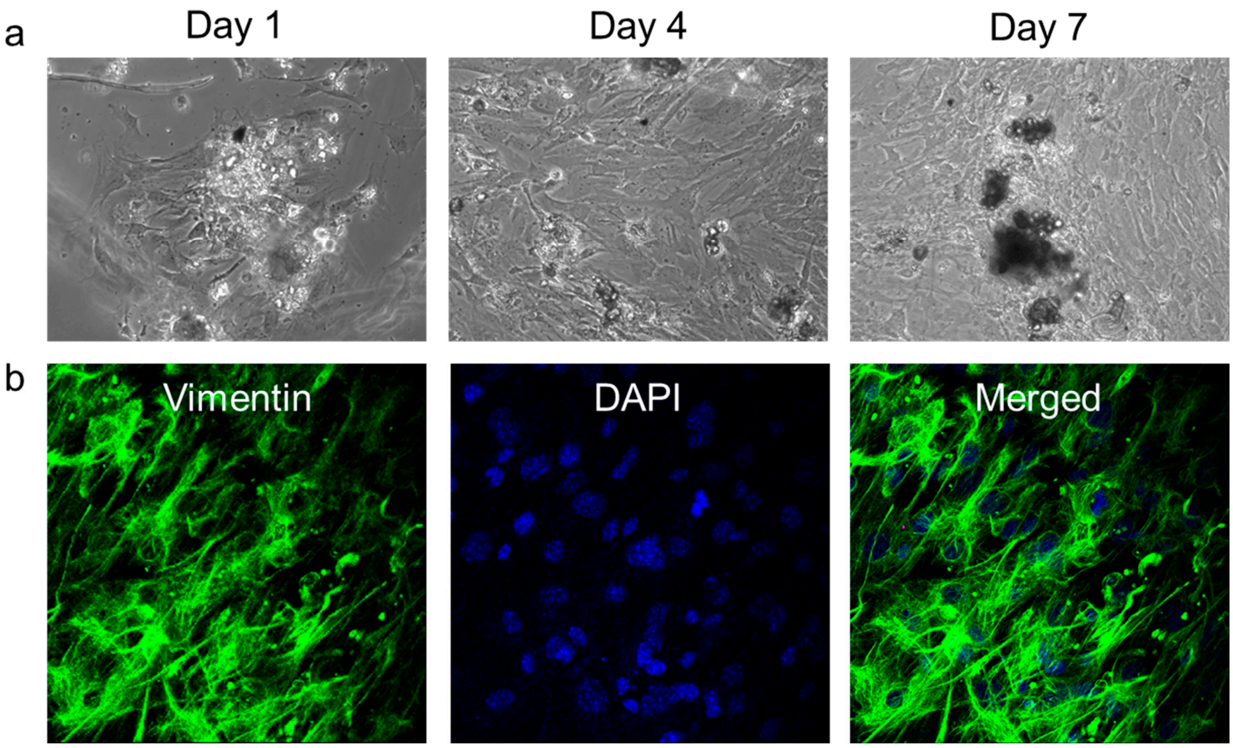

Figure S7. Images of primary E16 mesenchyme cells re-plated in a 6-well plate showing that these cells could proliferate after culturing in alginate hydrogel microtubes for 7 days followed by cell release from the microtube. (a) Optical images showing cell growth on days 1, 4 and 7. (Magnification: 20x) (b) Confocal images showing expression of vimentin after growth in a 6-well plate for 7 days. (Green: vimentin; Blue: DAPI-stained nuclei; Magnification: 63x).
